# Lead as an Abortifacient

**Published:** 1898-08-06

**Authors:** 


### LEAD AS AN ABORTIFACIENT.

A fatal case of lead-poisoning from the use of diachylon pills taken for the purpose of bringing on a miscarriage is reported by Dr. George F. Crooke.[1] The case when seen presented symptoms so much more formidable than those which are commonly observed in ordinary chronic lead-poisoning that we think it desirable to draw attention to the aspect which lead-poisoning may take on when it assumes the form of what is spoken of as lead encephalopathy, as it does under some circumstances. The patient was a young married woman 23 years of age. She was lying on the floor raving in maniacal delirium, struggling violently with those who were endeavouring to restrain her, and apparently writhing in great agony. Her seizures were paroxysmal, and her intelligence was quite gone. Her breath was fœtid, and exceedingly offensive; the tongue was dry, and coated with a greyish black fur; blackened sordes had collected about the teeth and gu[illegible] along the free border of which there was a distinct sl[illegible]ty-blue line. The patient soon lapsed into a condition of coma, on which convulsions of a violent nature supervened. She seemed blind, and on examination both optic discs were found to be quite choked. The urine when drawn off was found to be acid, contained albumin, leucocytes, and a few red blood-cells and cellular and hyaline casts. She died two days later, never having thoroughly regained consciousness. From the time that the coma and convulsions set in there was considerable rise of temperature. She had miscarried about a week before this condition of affairs came on, but on the post-mortem examination there was no evidence of inflammatory mischief about the uterus. Dr. Crooke says that the practice of taking diachylon in the form of pills, for the purpose of bringing on miscarriage, is far more prevalent among the working classes than is generally supposed, and that, in view of the known effects of lead upon the nervous system, the nature of some of the obscure nervous disorders, with attendant anæmia, which occur in women who have admitted miscarriage, may be cleared up by bearing in mind the possibility of lead-poisoning from the criminal use of diachylon. The less acute forms of lead-poisoning are so much the most frequent that we are all too apt to associate plumbism with colic or wrist-drop, or with early decrepitude and albuminuria, as it occurs, for example, among painters. The more serious forms, lead encephalopathy, in which convulsions and coma occur, leading rapidly to death, are rarely seen by the mass of practitioners; but such cases are known well enough to those who practise among operatives who are exposed to the more deadly forms of lead-poisoning. Not only, then, should the possibility of acute plumbism always be borne in mind, but it should be remembered that the onset of this form in young women is often masked by symptoms which appear to be of a hysterical nature, symptoms so slight, and apparently functional, as to be very apt to throw practitioners off their guard. Hysteria in a woman exposed to lead-poisoning is a matter to treat with much caution.

